# Molecular Basis Underlying Leaf Variegation of a Moth Orchid Mutant (*Phalaenopsis aphrodite* subsp. *formosana*)

**DOI:** 10.3389/fpls.2017.01333

**Published:** 2017-07-27

**Authors:** Chi-Chu Tsai, Yu-Jen Wu, Chiou-Rong Sheue, Pei-Chun Liao, Ying-Hao Chen, Shu-Ju Li, Jian-Wei Liu, Han-Tsung Chang, Wen-Lin Liu, Ya-Zhu Ko, Yu-Chung Chiang

**Affiliations:** ^1^Kaohsiung District Agricultural Research and Extension Station Pingtung, Taiwan; ^2^Department of Biological Science and Technology, National Pingtung University of Science and Technology Pingtung, Taiwan; ^3^Department of Food Science and Nutrition, Meiho University Pingtung, Taiwan; ^4^Department of Life Sciences and Research Center for Global Change Biology, National Chung Hsing University Taichung, Taiwan; ^5^Department of Life Science, National Taiwan Normal University Taipei, Taiwan; ^6^Department of Biological Sciences, National Sun Yat-sen University Kaohsiung, Taiwan; ^7^Department of Biomedical Science and Environment Biology, Kaohsiung Medical University Kaohsiung, Taiwan

**Keywords:** alternative polyadenylation, alternative splicing, chloroplast structure, differential expression protein, proteome analysis, variegation mutant

## Abstract

Leaf variegation is often the focus of plant breeding. Here, we studied a variegated mutant of *Phalaenopsis aphrodite* subsp. *formosana*, which is usually used as a parent of horticultural breeding, to understand its anatomic and genetic regulatory mechanisms in variegation. Chloroplasts with well-organized thylakoids and starch grains were found only in the mesophyll cells of green sectors but not of yellow sectors, confirming that the variegation belongs to the chlorophyll type. The two-dimensional electrophoresis and LC/MS/MS also reveal differential expressions of PsbP and PsbO between the green and yellow leaf sectors. Full-length cDNA sequencing revealed that mutant transcripts were caused by intron retention. When conditioning on the total RNA expression, we found that the functional transcript of *PsbO* and mutant transcript of *PsbP* are higher expressed in the yellow sector than in the green sector, suggesting that the post-transcriptional regulation of *PsbO* and *PsbP* differentiates the performance between green and yellow sectors. Because *PsbP* plays an important role in the stability of thylakoid folding, we suggest that the negative regulation of *PsbP* may inhibit thylakoid development in the yellow sectors. This causes chlorophyll deficiency in the yellow sectors and results in leaf variegation. We also provide evidence of the link of virus CymMV and the formation of variegation according to the differential expression of CymMV between green and yellow sectors.

## Introduction

Foliar variegation is found in various plants. This attractive trait often enhances the commercial value of ornamental plants. There are two major categories of mechanisms responsible for leaf variegation: chemical color- (pigment)-related variegation and physical color- (structural)-related variegation (Hara, 1957; Sheue et al., 2012). Chlorophyll-deficiency variegation is one of the most common chemical-color mechanisms occurring in ornamental plants, including in *Ficus* *pumila* ‘Sonny’ (Sheue et al., 2012). Pigmented-leaf variegation is derived from the formation of sectors that contain either normal-appearing chloroplasts or abnormal plastids, or other non-chlorophyll pigments (e.g., anthocyanin; Hara, 1957). Some variegated leaves of such mutants show various kinds of green and yellow sectors that are believed to originate from mutations in nuclear or organelle genes (Tilney-Bassett, 1975).

Thylakoids are the prominent structures inside mature chloroplasts. The formation of thylakoid membranes is closely related to the development of chloroplasts from undifferentiated proplastids. The thylakoid membrane-enclosed-pigment-protein complex, photosystem II (PSII), catalyzes the light-induced electron transfer from water to plastoquinone. This reaction occurs in the oxygen-evolving complex (OEC), which can be stabilized and protected by extrinsic membrane proteins attached to the lumenal side of the PSII. These extrinsic membrane proteins include PsbO (33 kD), PsbP (23 kD), and PsbQ (17 kD) and, in higher plants, are encoded by multigene families (Seidler, 1996; Lin et al., 2008). PsbO is the stable binder of the Mn cluster by facilitating Cl^-^ retention in PSII (Miyao and Murata, 1985; Roberts et al., 2012). Similarly, PsbP is involved in Ca^2+^ binding and supply, increasing the OEC affinity for Ca^2+^ and Cl^-^ (Ghanotakis et al., 1984), while PsbQ mainly participates in Cl_2_ retention (Miyao and Murata, 1985). Based on structural analyses of grana (i.e., a stack of thylakoids) in chloroplasts, Dekker and Boekema (2005) suggested that the interactions between the lumenal cofactors of the PSII complexes and thylakoid membranes are important for the development of grana. Suorsa and Aro (2007) found that most lumenal-exposed PSII proteins are PsbP and PsbQ and so proposed that these two proteins may contribute to the formation of the shape of grana.

Variegation is frequently attributed to transposons, but the most well-known variegation mutations can be divided into nuclear and plastid mutations. For example, in plastid mutations, the maize *IOJAP* and barley *albostrians* mutants are plastid defects lacking chloroplast ribosomes (Hess et al., 1994; Han and Martienssen, 1995). These mitochondrial mutations result in the variegation phenotype, a result of the maize non-chromosomal stripe mutant (NCS), which is caused by a mitochondrial defect (Roussell et al., 1991; Gu et al., 1993). Several nuclear variegation mutants are known in *Arabidopsis*, including *var1*, *var2*, *chloroplast mutator*, and *immutans* (Yu et al., 2007). Therefore, variegation mutants can provide a model system for studying the mechanisms of chloroplast biogenesis (Liu et al., 2010).

In this study, the cell structure and proteome of green and yellow sectors of leaves were compared in order to identify the regulatory mechanisms for variegation mutations in *Phalaenopsis aphrodite* subsp. *formosana*. Our results show that defects in the yellow sectors of mesophyll occurring during chloroplast development can cause chlorophyll accumulation. This result is consistent with comparative proteome data showing that the OEC protein PsbP is reduced in yellow sectors and is involved in chloroplast development. In addition, a differential protein expression between green and yellow sectors has also been shown under alternative polyadentylation of post-transcriptional gene regulation.

## Materials and Methods

### Plant Material

**FIGURE 1:**
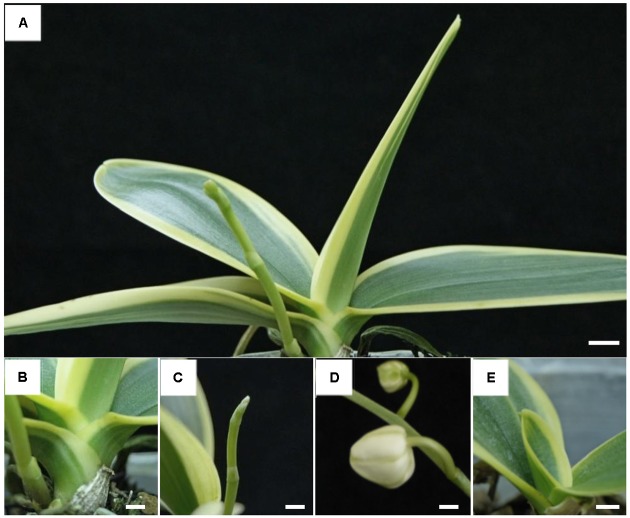
Plant phenotype of *Phalaenopsis aphrodite* subsp. *formosana* variegated line. **(A)** Plant, **(B)** stalk, **(C)** flower bud, **(D)** pedicle, and **(E)** young leaf. All scale bars: 1 cm.

The variegated mutant of *P. aphrodite* subsp. *formosana* (Orchidaceae) is an important native parent plant used for *Phalaenopsis* breeding (Tsai et al., 2014, 2015a,b; Liu et al., 2016). Ten individuals of this mutant were collected from fields and cultivated in the Kaohsiung District Agricultural Research and Extension Station, Taiwan (**Figure 1**).

### Light Microscopy and Confocal Laser Scanning Microscopy (CLSM)

The green and yellow sectors of each individual were observed with a light microscope (Leica DFC 480) and with a confocal laser scanning microscope (CLSM) (Leica CTR 6500) with excitation and emission wavelengths of 633 and 640–700 nm, respectively.

### Transmission Electron Microscopy (TEM)

Small leaf pieces (1.0 mm × 1.0 mm) were obtained from both green and yellow areas of each individual, then were cut and fixed in 2.5% glutaraldehyde in 0.1 M sodium phosphate buffer (pH 7.3) overnight at 4°C. These samples were then fixed in 1% OsO_4_ in 0.1 M sodium phosphate buffer for 4 h following an ethanol-series dehydration. These materials were then embedded in Spurr’s resin (DER = 6.0) (Spurr, 1969) and then polymerized at 70°C for 12 h. These samples were then cut into semi-thin sections (1 μm) with an MTX Ultramicrotome (RMC, Tucson, AZ, United States). For clearer observation under a light microscope (OLYMPUS BX-51, Tokyo, Japan), we stained these samples with 1% toluidine blue for 2 min. For further transmission electron microscopy (TEM) examination, ultrathin (70 nm) sections were cut and stained with 5% uranyl acetate (in 50% methanol) and 1% lead citrate (in water) and then examined using a TEM (JEOL JEM-1400, Tokyo, Japan).

### Preparation of Proteins and Two-Dimensional Electrophoresis

From each individual, green and yellow variegated leaf tissues (1 g from each region) were frozen in liquid nitrogen and ground on ice. The ground tissue was homogenized for 30 s in a solution containing 2.5 mL Tris (pH 8.8)-buffered phenol, 2.5 mL extraction buffer [0.1 M Tris-HCl (pH 8.8), 1 mM EDTA, 1 μg/mL leupeptin, 1 μg/mL pepstatin, 1 mM benzamidine, and 20 mM DTT], and a 25 μL protease inhibitor cocktail (Sigma). The homogenized tissue solution was transferred to a 15-mL centrifuge tube, shaken on ice for 30 min, and centrifuged for 30 min at 6000 *g* at 4°C. The upper layer (phenol phase) was collected as the first phenol fraction. The lower layer (aqueous phase) was then mixed with 2.5 mL of Tris-buffered phenol (pH 8.8) and a 2.5 mL extraction buffer, which was then incubated for 5 min on ice. The mixture was centrifuged for 10 min at 6000 *g* at 4°C, and then the phenol phase was collected and combined with the first phenol fraction. The combined phenol fractions were combined with a threefold volume 10% TCA/acetone solution (90% acetone, 10% TCA, 20 mM DTT) and incubated for 1 h at -20°C. The mixed solution was then centrifuged for 10 min at 1000 *g* at 4°C. Acetone was added to wash the precipitate and remove salt; this wash procedure was repeated three times. The precipitate was homogenized with 100 μL sample buffer [6 M urea, 2 M thiourea, 0.5% triton X-100, 10 mg/mL DTT and 5 μL IPG buffer (pH 4–7)] incubated for 3 h at 37°C, and centrifuged for 15–20 min at 20,000 *g* at 4°C.

Isoelectricfocusing (IEF) was carried out on 11-cm ReadyStrip IPG strip gels (BioRad) as well as on Immobiline DryStrip gels (GE Healthcare, Chalfont St. Giles, United Kingdom) at pH 4–7. The strips were allowed to rehydrate in a PROTEAN IEF Cell (BioRad) for 12 h at 50 V. Proteins were separated at pH 4–7 by IEF. The protocol used was 15 min at 250 V, 5 h at 4000 V, and a final program of 12 h at 50 V, 2 h at 100 V, 2 h at 500 V, 2 h at 1000 V, 2 h at 4000 V, and 3 h at 8000 V, until a total of at least 95,000 V was reached. Strips were then held at 500 V. After focusing, the strips were immediately run or frozen at -80°C. The IEF strip was washed with water to remove the mineral oil and then placed into 2.5 mL of equilibration buffer I [6 M urea, 375 mM Tris-HCl (pH 8.8), 2% SDS, 20% glycerol, 2% (w/v) DTT] and incubated for 15–20 min. Each strip was equilibrated in 2.5 mL of equilibration buffer II [6 M urea, 375 mM Tris-HCl (pH 8.8), 2% SDS, 20% glycerol, 2.5% (w/v) iodoacetamide] for 15–20 min, then applied to a 10% sodium dodecyl sulfate-polyacrylamide gel electrophoresis (SDS-PAGE) system (Mini-P III; Bio-Rad), and covered by 1% agarose containing bromophenol blue. The SDS-PAGE was performed at 100 V for 90 min after which the gel was stained with Coomassie Blue. The experiments were repeated three times for validation, and comparisons of protein expression were tested by two-sample *t*-test conducted with SPSS V21.0.

### Mass Spectrometry (MS) Analysis

The selected protein spots were cut and the proteins were reduced using 50 mM DTT at 37°C for 1 h. After removal of the supernatant, proteins were alkylated using 100 mM IAM at room temperature. After removing the supernatant, the gel was dehydrated using acetonitrile (ACN). Afterward, the dehydrated gel was lyophilized for 5 min under vacuum to remove the ACN. The lyophilized gel pieces were submersed in 25 mM ammonium bicarbonate and sequencing grade modified trypsin was added at an enzyme/protein ratio of 1/20 (w/w), then the mixture was incubated at 37°C for 16 h. The tryptic peptides were extracted using 50% ACN in 1% trifluoroacetic acid (TFA). The resulting peptide mixture derived from the in-gel digestion was separated using a Capillary HPLC system (Waters). This separation utilized a 75 μm i.d. capillary column packed with 5 μm particles (MST, Taiwan). This provided a gradient from 5 to 70% acetonitrile containing 0.1% formic acid over 75 min at a flow rate of 300 nL/min. The separated peptides were analyzed online on a Q-TOF mass spectrometer (Micromass, United Kingdom) equipped with a nano ESI source. The scan range was from 400 to 1600 m/z for MS and 50 to 2000 m/z for MS/MS. The MS/MS raw data were automatically processed into PKL files and the resulting files were searched using the Mascot search engine v2.2. The database search parameters were arranged as follows: (1) the protein database was set as NCBI; (2) the taxonomy was set as green plant; (3) the enzyme was set as trypsin; (4) one missed cleavage was allowed; (5) the precursor and product ion mass tolerance were set at 0.1 and 0.5 Da, respectively; (6) carbamidomethyl (C) was chosen for fixed modification; (7) oxidation (M) was chosen for variable modification; (8) proteins with scores above the significance threshold (*p* < 0.05) were shown as significant hits. The hit with the highest score was regarded as the identified protein from each gel spot.

### Rapid Amplification of cDNA Ends (RACE)

To isolate both *PsbO* and *PsbP* genes, the degenerated primers were separately designed to clone partial DNA fragments of *P. aphrodite* according to the conserved region of these two genes submitted in NCBI. After that, the full-length of these two genes were isolated by RACE analysis. A SMART RACE cDNA amplification kit (Clontech, United States) was used to obtain full-length transcripts and the sequences of *PsbO* and *PsbP* genes are available from GenBank (accession numbers: MF346691 for *PsbO* and MF346690 of *PsbP*). Total RNA was extracted using an RNeasy Plant Mini kit (Qiagen, Inc., Valencia, CA, United States), and the cDNA were synthesized as recommended by the SMART RACE kit. Gene-specific primers (GSP): PsbO (RAO1: 5′-TACCCAAGCGGCTGACCTTCGACG-3′), PsbP (RAP1: 5′-GTGGCTGGGGAGGGAGCATG-3′) and nested gene-specific primers (NGSP): PsbO (RAO2: 5′-ACGTATACGGAGGTGAAAGGGTCGGG-3′), PsbP (RAP2: 5′-ATAAGGCGATGAGACCTGGAG-3′) were designed for 5′- and 3′-RACE using the software primer premier 5.0. The primer sequences are listed in Supplementary Table S1.

The first round PCRs were performed with the GSP primer and a Universal Primer Mix (UPM). The 1/1000X-diluted first PCR products were used as the templates in the nested PCRs with NGSP. The RACE products were separated on an agarose gel and purified using the Gel-M^TM^ Gel extraction system kit (Viogene, Taipei, Taiwan). Purified cDNA was ligated into the pGEM-T Easy Vector (Promega, United States) and sequenced completely from both directions. All RACE results were confirmed by end-to-end PCRs.

### Gene Expression Analysis

Total RNAs were extracted from variegated leaves using an RNeasy Plant Mini kit (Qiagen, Inc., Valencia, CA, United States) followed by a DNase treatment (Qiagen, Inc., Valencia, CA, United States) following the manufacturer’s protocol, except that the length of incubation was 20 min. Reverse transcription (RT) was performed with reverse transcriptase (Promega, United States) using 500 ng of total RNA in a 10-μL reaction volume of which 0.5 μL of reaction solution was used as the PCR template for gene expression. The real-time PCR analysis used a 2X Fast SYBR green master mix (Applied Biosystems, United States) with the ABI 7900 PRISM Sequence Detection System. Semi-quantitative RT-PCR (qRT-PCR) measured the gene expression using specific primers (Supplementary Table S1) with 18S rRNA as the internal control. Thermocycling conditions were as follows: initial denaturation occurred at 94°C for 5 min, followed by 30 s (for *PsbP* and *PsbO*) of 20 s at 94°C, 20 s at 59°C, 30 s at 72°C, and terminated by a 7 min final extension at 72°C. Each experiment was repeated at least three times and each sample were collected from at least three individuals.

## Results

### Comparison of Chloroplast Structure between Green and Yellow Sectors Headings

**FIGURE 2:**
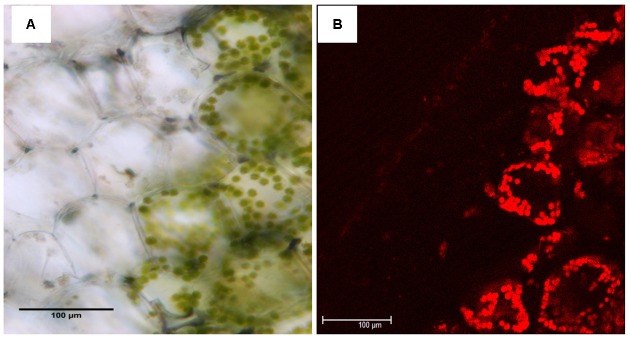
Comparison of leaf structure between the yellow (Y) and green sectors (G) of a variegated leaf of *P. aphrodite* subsp. *formosana*. Inspection of cross sections of yellow sectors using **(A)** light microscopy and **(B)** confocal microscopy does not reveal chloroplasts filled with chlorophyll. Scale bars: 100 μm.

**FIGURE 3:**
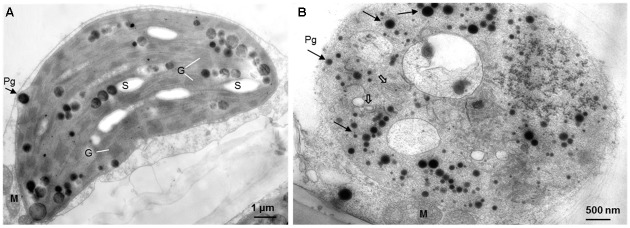
The comparative ultrastructure of plastids in a variegated leaf. **(A)** The green leaf area with typical chloroplasts, including well-organized thylakoids, grana, and starch grains. **(B)** The yellow leaf area with poor developed plastids, with few unstacked and loose thylakoids (open arrows), rich of plastoglobuli (arrows), but no starch grains. M: mitochondrion, G: granum, S: starch grain, Pg: plastoglobulus.

When observed with light and Confocal microscopy, normal chloroplasts (those filled with chlorophyll) were not found in the mesophyll cells of yellow sectors (**Figure 2**). Ultrastructure of chloroplasts revealed disk-like shape with an abundance of distinct thylakoids packed into grana containing many starch grains in cells of the green sectors under the observation of TEM (**Figure 3A**). In contrast, the plastids in cells of the yellow sectors stayed at a proplastid stage (the initial developmental stage of a chloroplast). These proplastids contained only unstacked and loose thylakoids, few in number, indicating they were poorly developed. Many of the plastids in the cells of the yellow sectors became vacuole-like and contained numerous plastoglobuli of various sizes (**Figure 3B**).

### Differential Protein Expression between Green and Yellow Sectors

**FIGURE 4:**
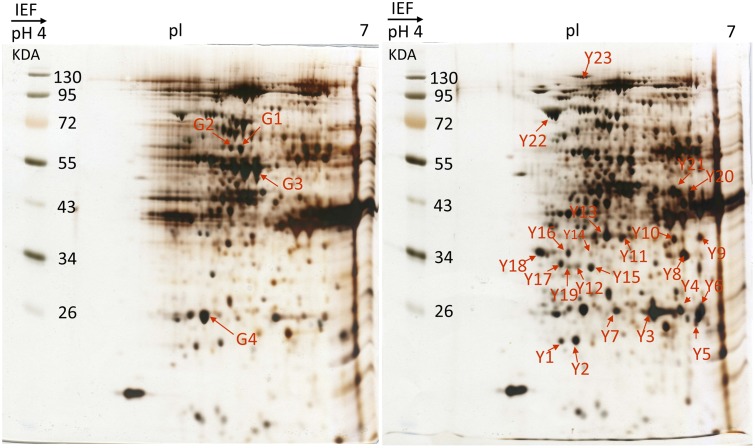
2-DE gel image of acidic range (pH 4–7) of total protein fraction from green sectors and yellow sectors. Two-dimensional electrophoresis analysis of green and yellow *P. aphrodite* proteins. Proteins were separated by gel electrophoresis followed by Coomassie blue staining. The pH and molecular weight standards are labeled. *PsbO* and *PsbP* spots are delineated by boxes. All experiments were repeated three times. pI, isoelectric points.

Differential protein expressions between the green and yellow sectors of leaves were identified via two-dimensional gel electrophoresis (2-DE) and mass spectrometry (MS). Several disparate spots were detected on 2-D gels within the pH range of pH 4–7 and a molecular weight (MW) range of 17–130 kDa (**Figure 4**). Of these, 27 spots of differential proteins, including four down-regulated and 23 up-regulated expressions in yellow sectors, were cut from 2-DE. These protein IDs were identified using MS followed by a database search. From this, 13 spots of differential proteins were identified. One down- and 12 up-regulated expression proteins were identified in the yellow sectors. The down-regulated protein was PsbP, while the 12 up-regulated proteins belonged to seven categories: PsbO, myosin-like protein, actin, the Cymbidium mosaic virus (CymMV) coat protein, aspartate aminotransferase 2 precursor (AAT2) protein, glyceraldehyde-3-phosphate dehydrogenase (GADPH), and chloroplast heat shock protein 70-1 (CPHSC70-1) (**Table 1**). Both PsbP (23 kDa) and PsbO (33 kDa) are involved in the OEC and will be detailed in another study. These results were consistent in all three replicate samples (**Supplementary Figure S1**). Among these 13 spots, eight spots reveal significantly differential protein expressions, including one down-regulated protein (PsbP) and seven up-regulated proteins (**Supplementary Figure S2**). Both qRT-PCR and real-time PCR were conducted to validate the differential protein expressions of the three proteins.

**Table 1:** Differential accumulation of proteins between green and yellow sectors in variegated leaves as revealed by mass spectrometry analysis.

Spot no.	Protein	Reference organism	Accession no.	Calculate-Mr/pI	Percentage covered (%)	Score	Match peptide
G1	PsbP	*Arabidopsis thaliana*	PA0013	1434/9.71	92	94	18
Y1	Similar to myosin protein	*Bos taurus*	XP_612582	227062/5.5	3	123	4
Y4	Actin	*Brachionus plicatilis*	CAA44532	27248/5.35	15	107	2
Y5	Actin A3	*Bombyx mori*	CAA28192	41865/5.47	5	88	2
Y7	CymMV coat-protein	*Cymbidium mosaic virus*	AAQ62703	23765/6.15	15	185	9
Y13	Actin	*Haliotis rufescens*	AAB87082	17931/5.43	17	87	1
Y14	PsbO	*Fritillaria agrestis*	O49079	34848/6.26	9	119	4
Y15	PsbO	*Fritillaria agrestis*	O49079	34848/6.26	8	109	5
Y16	Actin-like protein	*Homo sapiens*	AAX82281	11479/5.39	33	138	2
Y17	Actin	*Lepeophtheirus salmonis*	ABU41077	41727/5.37	8	119	2
Y21	AAT2	*Canavalia lineata*	AAB68396	50936/8.56	3	79	2
Y22	GADPH	*Hordeum vulgare*	P08477	33215/6.20	12	93	5
Y23	CPHSC70-1	*Arabidopsis thaliana*	NP_194159	76461/5.07	7	251	11

### Full-Length Cloning of PsbO and PsbP Genes

**FIGURE 5:**
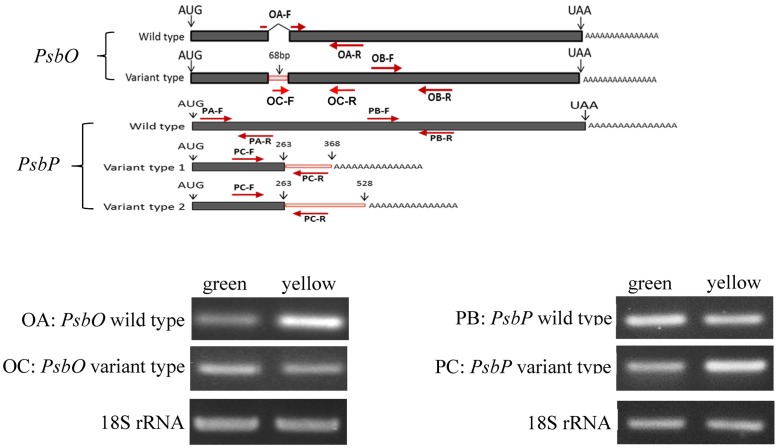
Reverse transcription PCR (RT-PCR) analyses of PsbP and PsbO polymorphic and 18S rRNA gene products of total RNA from green and yellow sectors of *P. aphrodite* subsp. *formosana*. Green: green sector; yellow: yellow sector; PA: all type; PB: PsbP wild-type; PC: PsbP mutant type; OA: wild-type; OB: all type.

There were no nucleotide differences between two *PsbO* transcripts except length variation. Such length variation with no nucleotide differences was found in cells of both the green and yellow sectors. The wild-type transcript was 1186-nt and 337 aa with 23-nt 5′ untranslated and 152-nt 3′ untranslated regions. The mutant transcript had a total length of 1254-nt with an additional 68-nt intron retention (**Figure 5**). In contrast, there was an additional stop codon within the intron of the mutant leading to an advance stop translation.

Similarly, there were three transcripts of *PsbP* with length variation but no nucleotide differences in both the green and yellow sectors. The wild-type transcript was 999-nt. The other two mutant transcripts have a 263-nt partial 5′ exon and a 105-nt intron at the 3′ end (mutant type 1) and a 263-nt partial 5′ exon with a 265-nt intron at the 3′ end (mutant type 2). Alternative polyadenylation (APA) was suggested to occur within the intron 1 region of *PsbP* and lead to two mutant transcripts of *PsbP* (**Figure 5**).

### Differential Display of PsbO and PsbP between Green and Yellow Sectors

**FIGURE 6:**
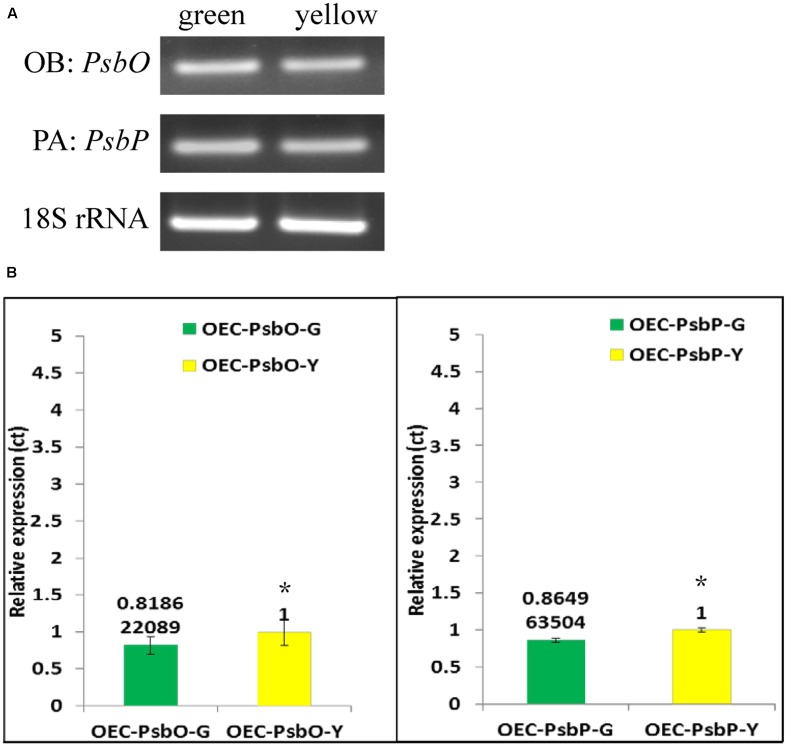
Reverse transcription-PCR **(A)** and Real-time PCR **(B)** analysis of gene expression in *PsbP*, *PsbO* and 18S rRNA gene products of total RNA from green and yellow sector of variegated leaf from *P. aphrodite* subsp. *formosana.* PA: all type; OB: all type. ^∗^*p* < 0.05 compare between green and yellow sectors.

**FIGURE 7:**
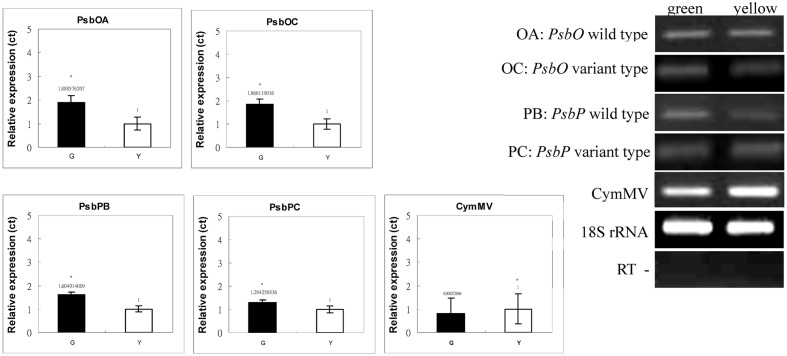
Real-time PCR analyses of PsbP and PsbO polymorphic and 18S rRNA gene products of total RNA from green and yellow sectors of *P. aphrodite* subsp. *formosana*. Green: green sector; yellow: yellow sector; PB: PsbP wild-type; PC: PsbP mutant type; OA: PsbO wild-type; OC: PsbO mutant type; CymMV: *Cymbidium* mosaic virus. ^∗^*p* < 0.05 compare between green and yellow sectors.

We used qRT-PCR and real-time PCR for differential displays of the *PsbO* and *PsbP* genes. Because the transcripts were not homogeneous, primers were designed for various gene regions to differentiate these genes: primers OB-F/R for amplifying both wild-type and mutant transcripts of *PsbO*; primers OA-F/R for only the wild-type transcript of *PsbO* (**Figure 5**) (Supplementary Table S1). Using primers OB-F/R for qRT-PCR and real-time PCR, we found no significant differences expression between the green and yellow sectors (**Figure 6**). Primers OC-F/R were also designed to detect the intron retention of mutant transcripts (**Figure 5**). Using primers specific for either wild-type or mutant-type *PsbO* transcripts, the gene expression of green sectors were shown to be prominently higher than that of the yellow sectors: 1.89-times higher in the wild-type and 1.87-times higher in the mutant-type (**Figure 7**). Our results show that the differential expression of *PsbO* between green and yellow sectors is not at the transcriptional level, but rather suggests alternative splicing (AS).

For the *PsbP* gene, primers PA-F/R were designed for amplifying both wild-type and the two mutant transcripts (mutant types 1 and 2); primers PB-F/R were designed specifically for the wild-type transcript; primers PC-F/R were specifically for the two mutant transcripts (**Figure 5** and Supplementary Table S1). Using primers PA-F/R, we found no significant differential expression in *PsbP* between green and yellow sectors (**Figure 6**). Using primers PB-F/R specific for the wild-type *PsbP* transcript, the gene expression of green sectors was prominently higher (by roughly 1.60 times) than that of yellow sectors (**Figure 7**). In contrast, using primers PC-F/R specific for both mutant types, the relative level of gene expression in the yellow sectors, despite being higher than the green sectors, was reduced by 1.28 times (**Figure 7**). These results show that the differential expression ratios of *PsbPB* and *PsbPC* in green and yellow sectors are due to APA rather than transcriptional differentiation. Based on our differential RNA expression analyses for both *PsbO* and *PsbP* genes, the differential expression of proteome between green and yellow sectors appears to be under post-transcriptional regulation, including AS and AP.

## Discussion

Our TEM revealed a strong contrast in chloroplast ultrastructure between green and yellow sectors of *P. aphrodite* subsp. *formosana* plant tissue. The plastids of the green sectors are functional chloroplasts retaining well-organized thylakoids, grana, and stroma thylakoids, with the presence of starch grains and bilayered membranes. In contrast, the yellow sectors have no normal chloroplasts. Rather, only proplastid-like or chromoplast-like plastids were found in the yellow sectors. Obviously, these yellow-sector plastids lack thylakoid and starch grains, but do contain numerous electron-dense plastoglobuli in the stroma with occasional incomplete, bilayered membranes. These results are similar to the chloroplast ultrastructure found in albino leaf sectors of variegated peach plants induced by a specific viroid RNA (Rodio et al., 2007) and albino mutants of bamboo (Lin et al., 2008), barley (Dunford and Walden, 1991), and rice (Gong et al., 2013). Such chlorophyll type variegation is a common condition in ornamental plant cultivars, but rarely occurs in wild plants. However, these plants with chlorophyll type variegation may be functionally deficient in photosynthesis due to the absence of functional chloroplasts in some leaf sectors (Sheue et al., 2012).

Previous studies have shown that a chlorophyll-deficient rice mutant, with reduced chlorophyll and abnormal chloroplast development, was caused by a mutant gene *yellow-green leaf 1* (*ygl1*) (Wu et al., 2007). The chlorophyll deficiency may be due to the block of chlorophyll synthesis or by an increase in the chlorophyll degradation rate (He et al., 2006). Such a chlorophyll deficiency can cause structural changes in a chloroplast’s development (He et al., 2006; Guo et al., 2007). Therefore, the chlorophyll synthesis and chloroplast development may be interdependent (von Wettstein et al., 1971). Our results show that only the mesophyll cells in the green sectors of variegated leaves of *Phalaenopsis* contain the complete chloroplast structure. Those cells in the yellow sectors have proplastids or chromoplast-like plastids filled with plastoglobuli (**Figures 2**, **3**). According to our proteomic analysis, no chlorophyll biosynthesis-related proteins were produced (**Figure 4**). Therefore, the yellow sectors of this variegated mutant are a consequence of the extremely reduced accumulation of chlorophyll in the yellow sectors, which leads to defective chloroplast development.

In tobacco, the PSII is hypersensitive to light without PsbP, and a rapid inactivation occurs if the repair process of damaged PSII is inhibited (Ifuku et al., 2005). Moreover, the manganese cluster is unstable in PsbP-deficient leaves. However, such phenotype alteration does not occur in the PsbQ-deficiency mutant of tobacco (Ifuku et al., 2005; Ifuku, 2014). These experiments indicated an essential function of *PsbP* is required for full PSII functioning, while *PsbQ* gene is not necessary (Ifuku et al., 2005; Ifuku, 2014). PsbP has been suggested to be functionally relative to Ca^2+^ binding and for increasing the affinity of the OEC for Ca^2+^ and Cl^-^ (De Las Rivas et al., 2007). In addition, PsbP could bind Mn^2+^ and act as a reservoir for binding and delivering manganese to the OEC (Bondarava et al., 2005).

On the basis of comparative proteomic analysis, it was difficult to predict whether the yellow sector relative to the green sector has significantly up-regulated protein expression. Of the differential protein expression shown in this study, PsbP, PsbQ, and CymMV coat proteins were particularly significant in the variegation phenotype, while PsbO was up-regulated in the yellow sectors. Previous studies of several albino plants show that PsbO was absent or down-regulated (Pérez-Bueno et al., 2004; Lin et al., 2008), which is inconsistent with the up-regulation control seen in the yellow sectors of the variegated mutant in this study. The pre-mRNA splicing and polyadenylation could affect the structure and expression of mature mRNA of eukaryotes (Proudfoot et al., 2002). The AS can produce multiple mRNAs from a single gene (Clower et al., 2010; David et al., 2010). Furthermore, APA is an important mechanism that generates a diversity of mature transcripts mediated by selecting an alternating site for poly(A) tail additions. Alternative sites could be located at different 3′ UTR coding regions, or introns of pre-mRNA (Shen et al., 2008; Xing and Li, 2011).

In this study, the AS and APA were found in *PsbO* and *PsbP* pre-mRNA, respectively, according to the RACE analysis for isolating full-length *PsbO* and *PsbP* genes in the green and yellow sectors. A partial intron fragment containing a stop codon was retained in the mutant type of *PsbO*, resulting in interrupted translation of a mutant transcript. In contrast, two mutant types of *PsbP* separately added poly(A) tails in different intron sites leading to two abnormal proteins being translated. Therefore, AS and APA can regulate *PsbP* and *PsbO* of pre-mRNA, respectively. *PsbP* and *PsbO* are differentially expressed in green and yellow sectors of variegated leaves. These results suggest that polyadenylation and splicing have a mechanistic interplay in the coupling of 3′ end processing and splicing as proposed by Millevoi et al. (2006).

We found no differential RNA expression in either *PsbO* or *PsbP* between green and yellow sectors using real-time PCR. However, the mutant transcripts derived from AS and APA caused differential PsbO and PsbP protein expression between the green and yellow sectors. According to transcript-specific PCR analysis, the wild-type transcript of *PsbP* was higher in the green sectors than the yellow sectors whereas the two mutant transcripts of *PsbP* were lower in the green sectors than the yellow sectors. In contrast, the mutant type of *PsbO* (derived from intron retention) was higher in the green sectors than the yellow sectors. These results indicates that pre-mRNA *PsbO* and *PsbP* were regulated by AS and APA, respectively. This transcriptional regulation is probably related to the variegation. To the best of our knowledge, there are very few examples of alternative spliced genes that can address gene regulation for a specific biological function. These include the AS of the *FCA* transcript, which has functional significance related to its role in the promotion of floral transition (Macknight et al., 2002) as well as stress-induced AS that results in greater accumulation of miR400 primary transcripts (Yan et al., 2012).

Virus infection could cause either up- or down-regulated protein expressions in plants and lead plants’ phenotype change (Takahashi et al., 1991; Rodríguez et al., 2012). However, it is difficult to differentiate genetic variegations from those induced by plant viruses because they are morphologically similar (Valverde et al., 2012). Examples of virus-induced variegation include abutilon infected by abutilon mosaic virus (AbMV) (Keur, 1934; Valverde et al., 2012), honeysuckle infected by honeysuckle yellow vein mosaic virus, and *Salvia splendens* infected by clerodendron golden mosaic China virus (Valverde et al., 2012). Similarly, the phenotype of *Phalaenopsis* species infected with a virus usually shows irregular chlorosis on the leaves (Zheng et al., 2011).

In this study, we found that the CymMV coat protein was expressed significantly higher in the yellow sectors than in the green sector. Therefore, we inferred that the phenotype of variegated mutants described in this study may relate to the CymMV infection despite no clear-cut evidence to support the direct effect of CymMV on variegation due to no inoculation experiment. Nevertheless, several studies have identified the responses of virus infection in plants and show various effects on components of the OEC. These viruses reduce the expression of PsbP (Kong et al., 2014), PsbO (Rodríguez et al., 2012), or both PsbP and PsbQ (Takahashi et al., 1991; Rahoutei et al., 2000; Sui et al., 2006), along with the appearance of chlorotic spots on leaves (Takahashi et al., 1991; Rahoutei et al., 2000; Sui et al., 2006; Rodríguez et al., 2012). OEC is one of the major protein targets in plants that interact with CymMV virus (Nanda and Biswal, 2008; Kong et al., 2014). Both alternating spliced mRNA and alternating polyadenylation in the intron of pre-mRNA was found in host cells infected with a virus (Verma et al., 2010). In this study, both AS and APA was suggested to be induced by the CymMV virus, which regulated *PsbP*, *PsbO*, and other genes. Of these, *PsbP* plays a key role in developing the variegated phenotype. Nevertheless, the CymMV virus must not be the original switch because the variegated phenotype differs between virus-infected plants and the variegated mutants studied here.

In this paper we present genetic, transcriptional and translational differences to figure out the molecular mechanism of PsbP on variegation. Our data showed that the AS and functional deficiency of PsbP primarily mediate the formation of variegation in *P. aphrodite* subsp. *formosana*. In the past, understanding of molecular mechanisms involved in the formation of variegation was very limited. Although this study still fails to answer all questions, a major step forward has been made in understanding the molecular regulation on variegation of orchid leaves.

## Author Contributions

Conceived and designed the experiments: C-CT and Y-CC. Performed the experiments: C-CT, Y-JW, C-RS, P-CL, and Y-CC. Analyzed the data: C-CT, Y-HC, S-JL, J-WL, H-TC, and Y-CC. Contributed reagents/materials/analysis tools: C-CT, Y-HC, S-JL, J-WL, H-TC, W-LL, Y-ZK, and Y-CC. Wrote the paper: C-CT, Y-JW, C-RS, P-CL, and Y-CC. Conceived of the study, edited the manuscript, and approved the final manuscript: C-CT, Y-JW, C-RS, P-CL, Y-ZK, and Y-CC.

## Conflict of Interest Statement

The authors declare that the research was conducted in the absence of any commercial or financial relationships that could be construed as a potential conflict of interest.

## Supplementary Material

FIGURE S1 Three different replications of 2-DE gel image of acidic range (pH 4–7) of total protein fraction from green sectors and yellow sectors.

FIGURE S2 Thirteen spots were cutted from three different replications of 2-DE gel image of acidic range (pH 4–7) of total protein fraction from green sectors and yellow sectors. These spots were processed for comparisons of protein expression using ImageJ and were tested by two-sample *t*-test conducted by SPSS V21.0. ^∗^*p* < 0.05 compare between green and yellow sectors.
