# Supplementary figures and images for: Molecular Basis Underlying Leaf Variegation of a Moth Orchid Mutant (Phalaenopsis aphrodite subsp. formosana)
Source: Front Plant Sci. 2017 Jul 27;8:1333. doi: 10.3389/fpls.2017.01333 (PMC5529386; doi:10.3389/fpls.2017.01333)

Repeat 1

Repeat 2

Repeat 3

G

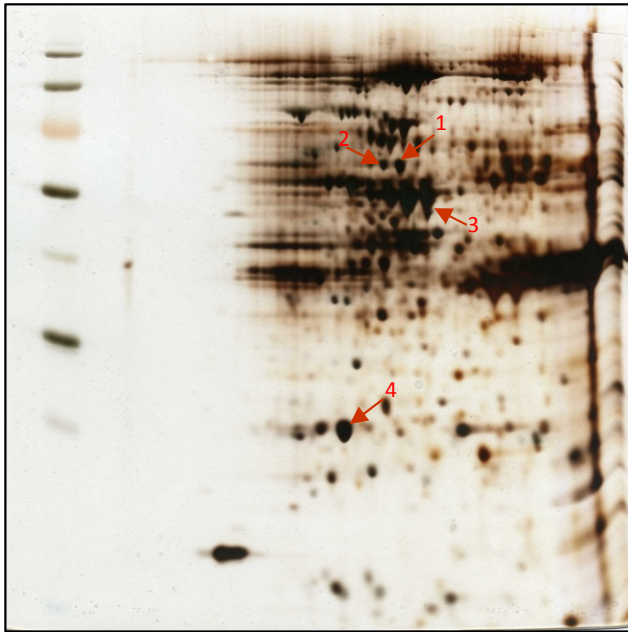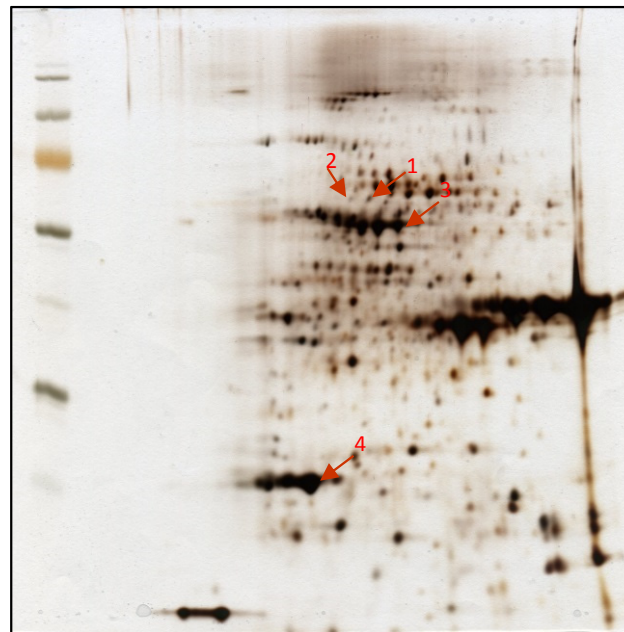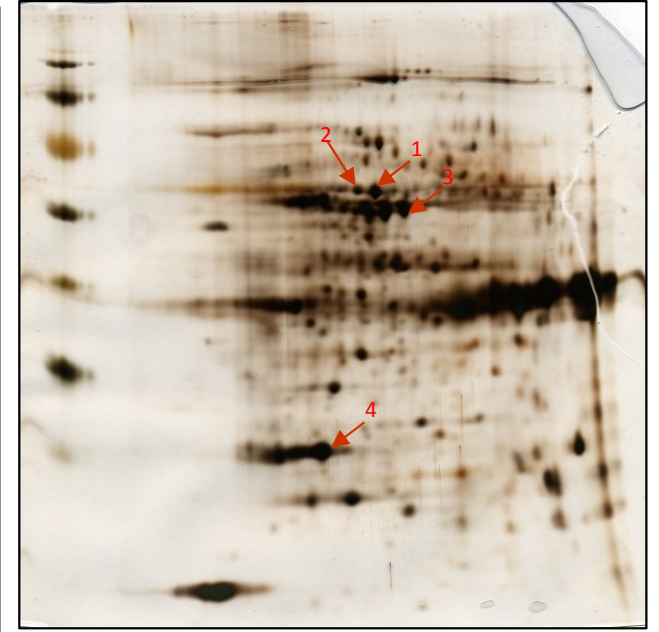

Y

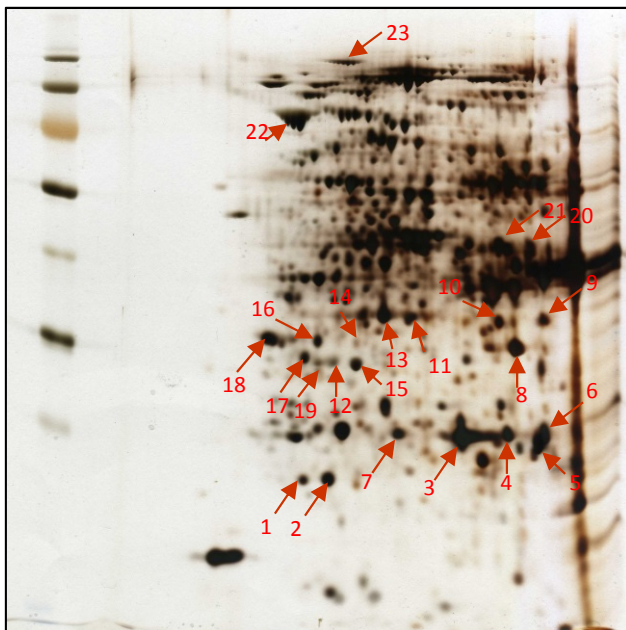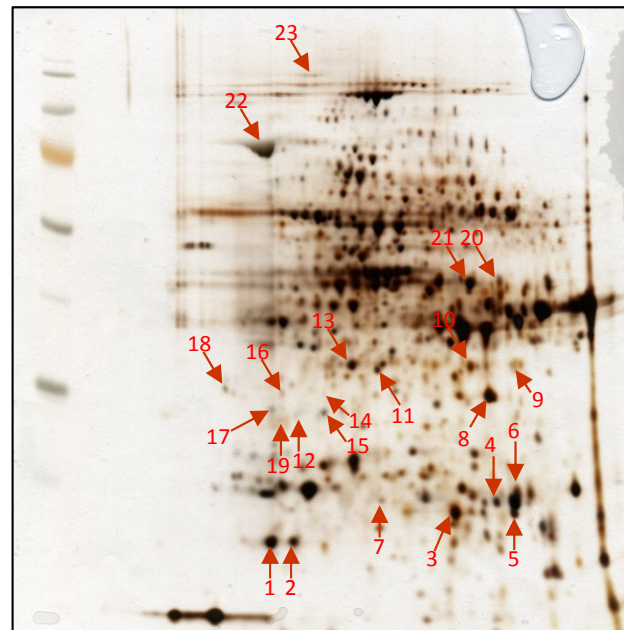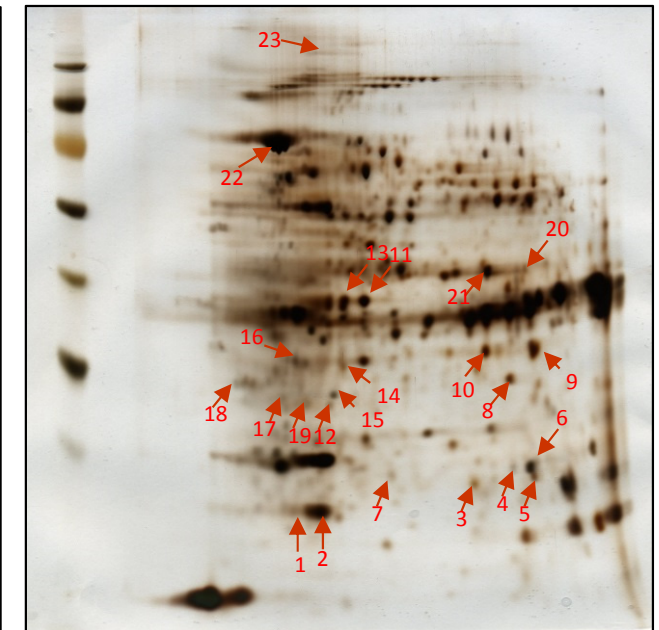

11 cm, pH 4-7, 12.5% SDS-PAGE

Supplement: FIGURE S1 — Three different replications of 2-DE gel image of acidic range (pH 4–7) of total protein fraction from green sectors and yellow sectors. [file Image_1.PDF]

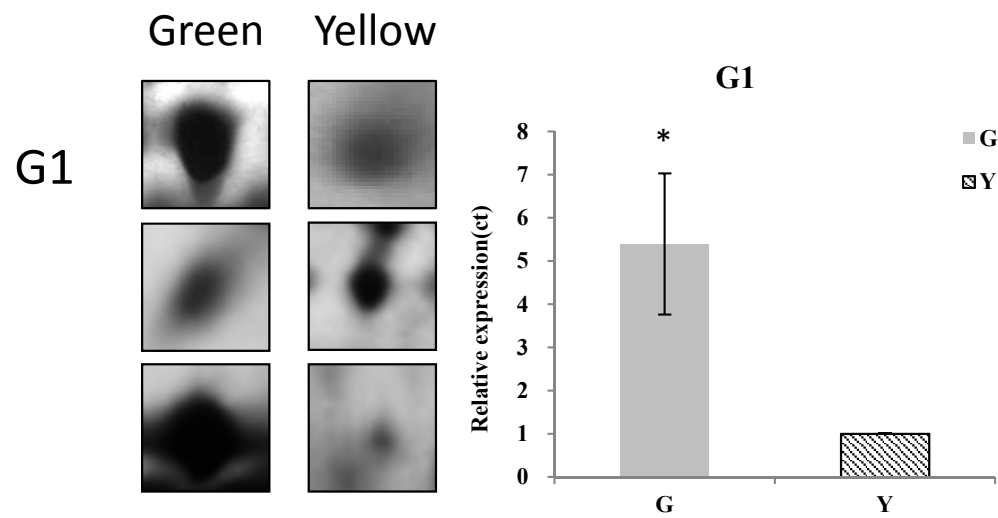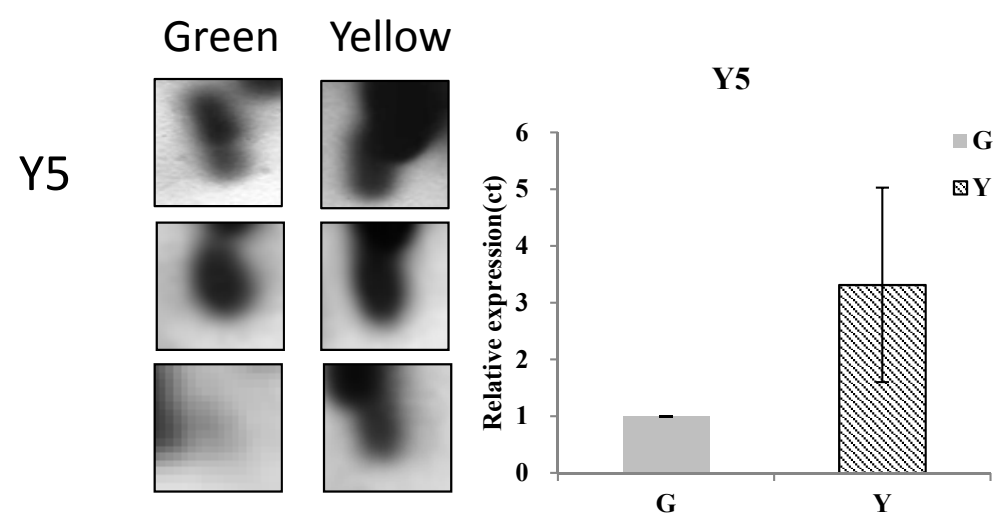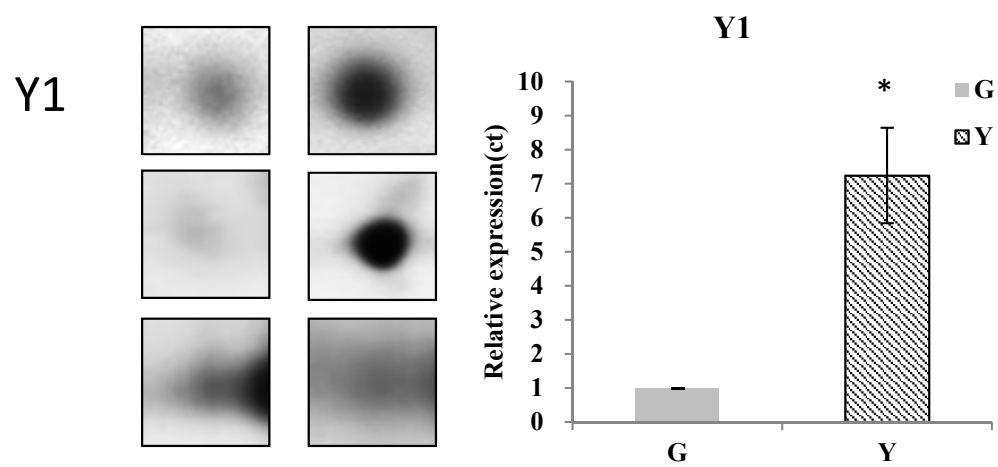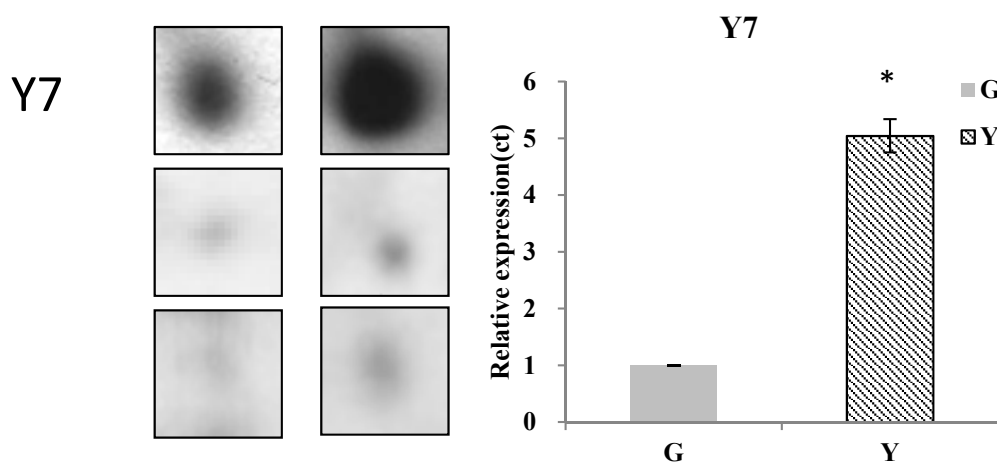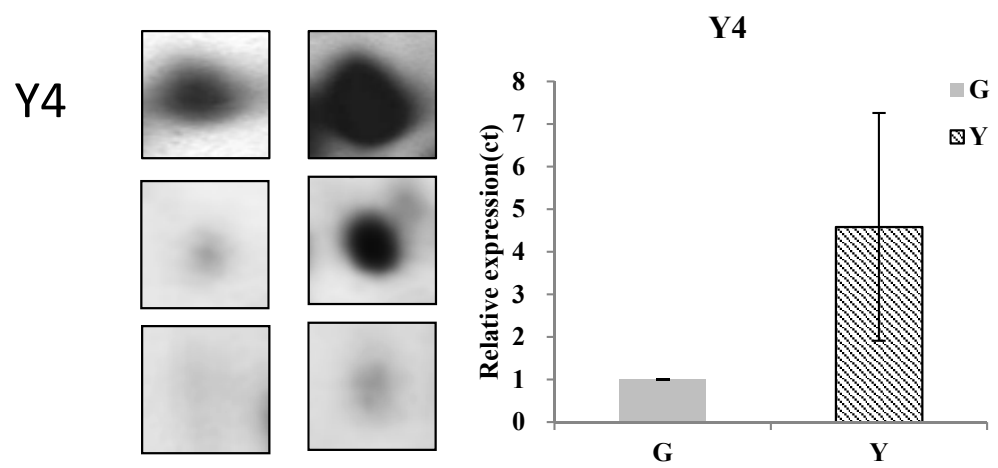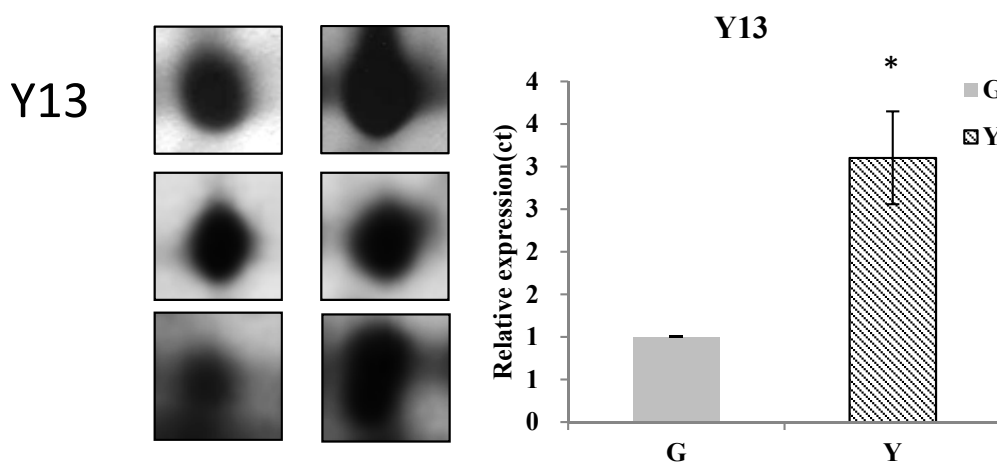

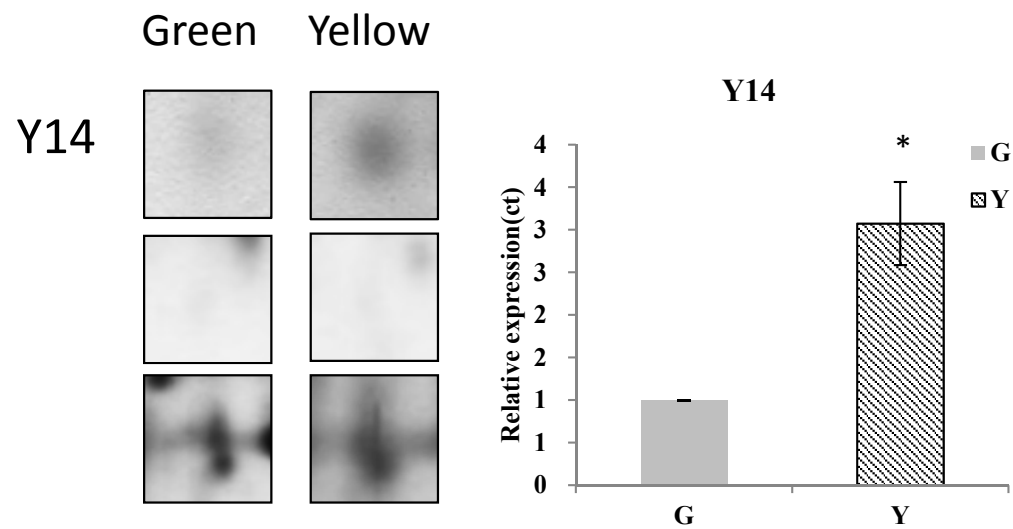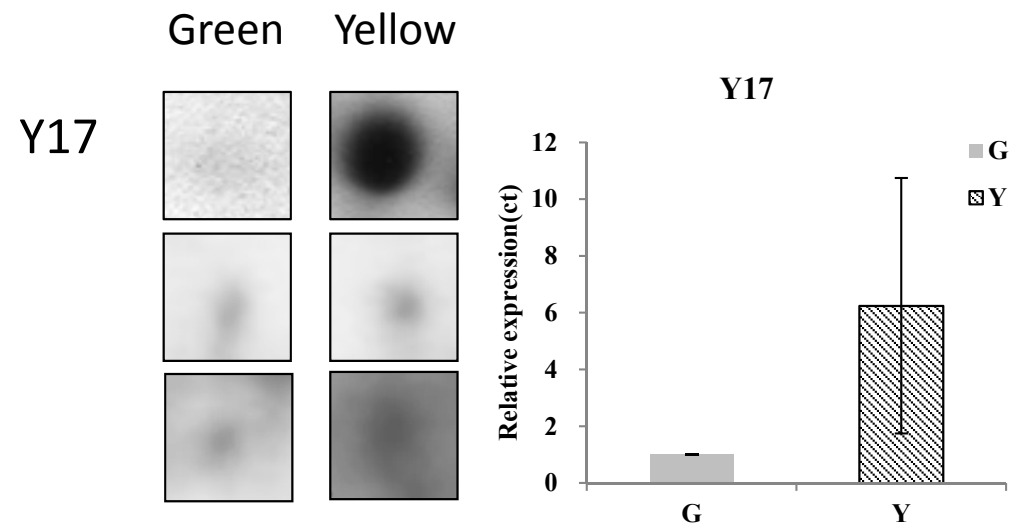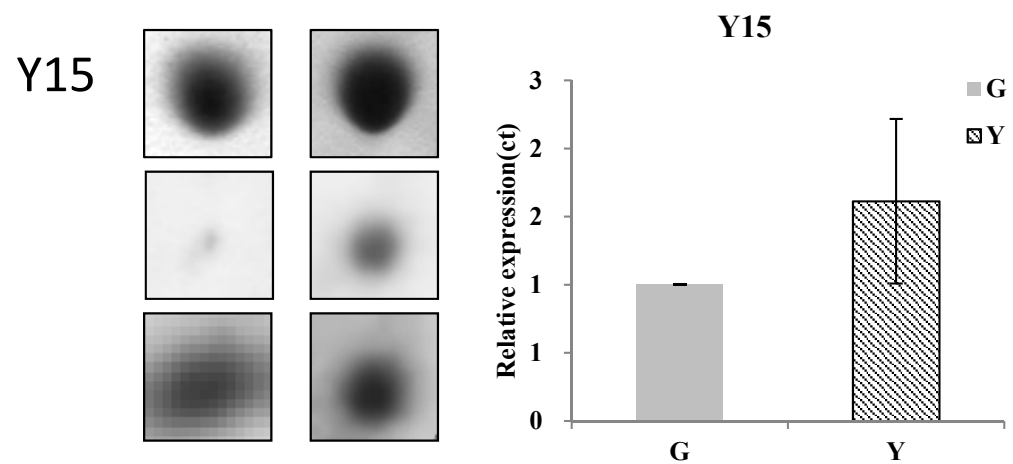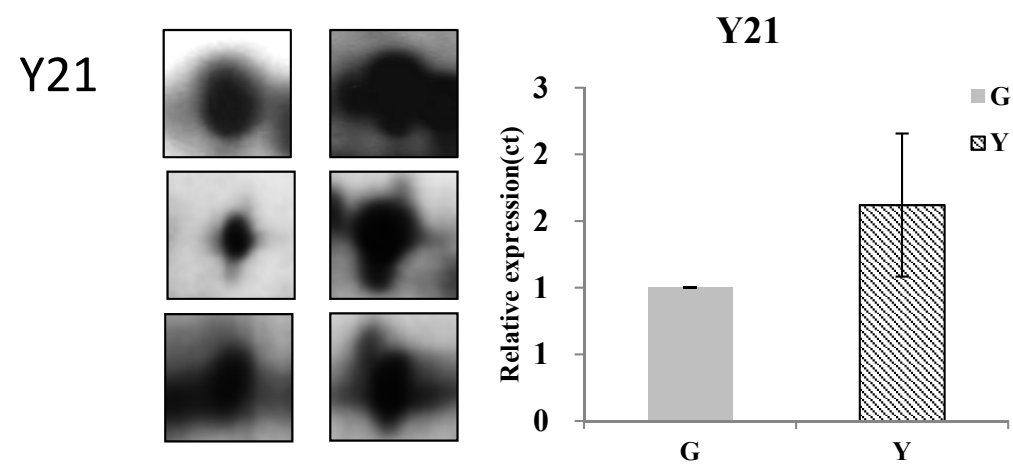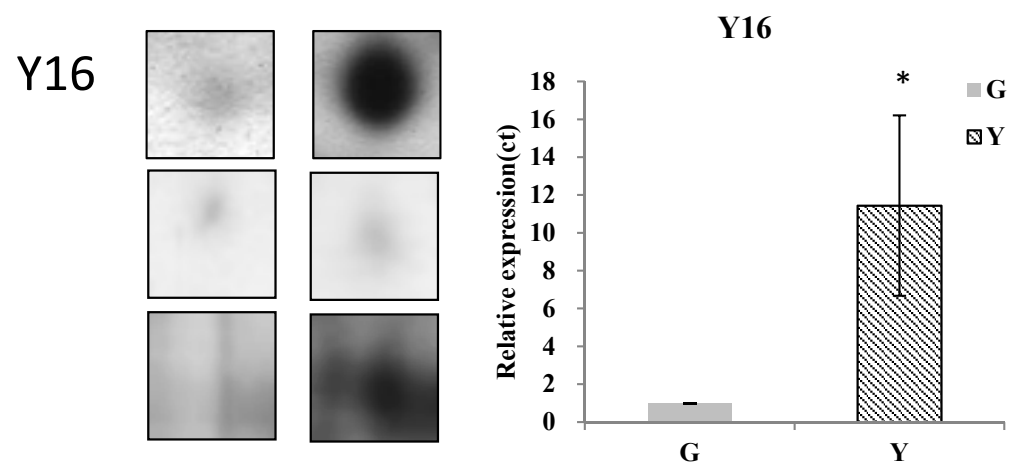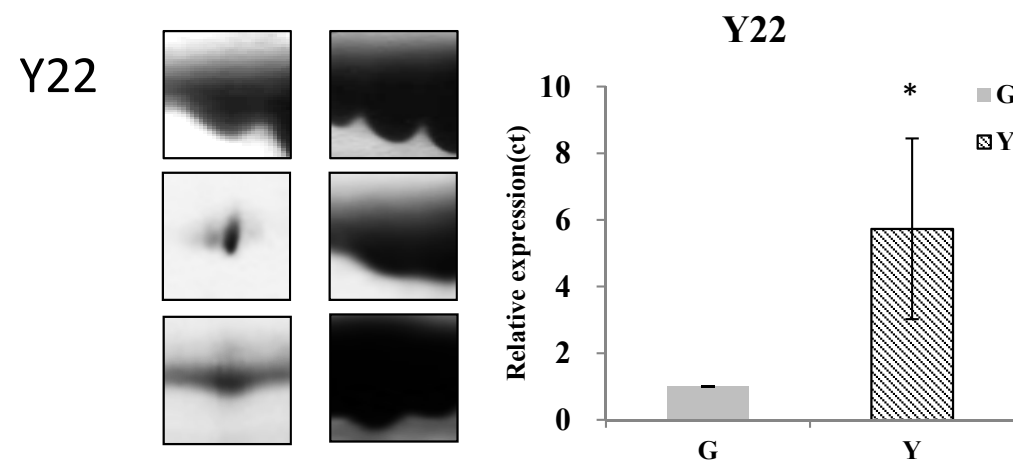

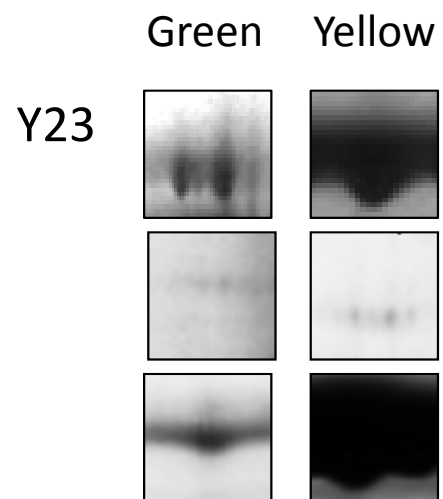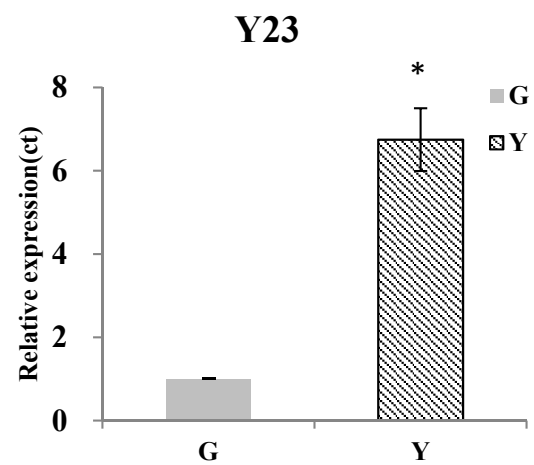

Supplement: FIGURE S2 — Thirteen spots were cutted from three different replications of 2-DE gel image of acidic range (pH 4–7) of total protein fraction from green sectors and yellow sectors. These spots were processed for comparisons of protein expression using ImageJ and were tested by two-sample t-test conducted by SPSS V21.0. ∗p < 0.05 compare between green and yellow sectors. [file Image_2.PDF]
