# Supplementary material for: Molecular Basis Underlying Leaf Variegation of a Moth Orchid Mutant (Phalaenopsis aphrodite subsp. formosana)
Source: Front Plant Sci. 2017 Jul 27;8:1333. doi: 10.3389/fpls.2017.01333 (PMC5529386; doi:10.3389/fpls.2017.01333)
Supplement: Supplementary file 3 [file Table_1.DOCX]

Supplementary Table 1. List of primers and their sequences used in the experiments.

| **Primer** | **Sequence 5’→3’** |
| --- | --- |
| OA-F | TCCTCGTCGCGGGCGCGAA |
| OA-R | AGTACTTACCCGGCTTGAAGGCGAA |
| OB-F | AAGAAGCTGTGCCTTGAGCCCAC |
| OB-R | TCGTCGAGTGTGTAGGTGAGTCTGG |
| OC-F | CTTCTTTCACTTAACCTAAGTAAAAGATGGC |
| OC-R | ATGGTCGGACACTGGTTCGCAGTTCC |
| PA-F | TTATGCCGAACCTCCGCTCTACC |
| PA-R | TGACGGCCAATCTGCGTGAGACAG |
| PB-F | CCAGCTCTTGATCCCATCCAAATGGAAC |
| PB-R | CATAGCTGTTGGAGTCAAAGTTGTCCTC |
| PC-F | AGCCGTGGTTGAGGAGAACAACTCTG |
| PC-R | GTAGTCGTTAAATCTTACCGGCTTCTC |
| 18S-F | ACATCTAAGGGCATCACGGACC |
| 18S-R | CTATGGGTGGTGGTGCATGGC |
